# Beyond usual suspects: phytobezoar in pediatrics a diagnostic challenge case report

**DOI:** 10.1097/RC9.0000000000000198

**Published:** 2026-03-03

**Authors:** Sergio David Angulo, Ángela María Giraldo, Andrés Sáenz Pinto, Karla Ortiz Vazquez, Sofía Mejía, María Silvana Rodriguez

**Affiliations:** a Caja de compensación familiar de Risaralda - Salud comfamiliar, Pereira, Risaralda, Colombia; b Universidad Tecnológica de Pereira, Pereira, Risaralda; c Colombian Society of Pediatrics, Pereira, Risaralda

**Keywords:** bezoar, phytobezoar, small bowel obstruction

## Abstract

**Introduction and importance:**

Bezoars are rare masses of indigestible material that accumulate in the gastrointestinal tract. The clinical presentation varies depending on the location and severity of the obstruction. Their true incidence remains unknown.

**Case presentation:**

A 17-year-old female adolescent with a history of dysfunctional home environment and chronic eating disorder presented with atypical, persistent abdominal pain and oral intolerance. Despite multiple diagnostic studies, including a diagnostic laparoscopy with no pathological findings, her condition progressively worsened, leading to septic shock. An emergency laparotomy revealed a terminal ileum perforation caused by a foreign body identified as a phytobezoar.

**Clinical discussion:**

This case highlights the diagnostic challenge of bezoars due to their nonspecific symptoms. Although computed tomography is the preferred imaging method, it may not be conclusive, as phytobezoars radiolucent nature may resemble fecal small bowel contents on CT scans.

**Conclusion:**

Bezoars should be included in the differential diagnosis for patients with atypical and persistent abdominal pain, even after multiple negative diagnostic tests. A high index of suspicion is crucial in patients with erratic symptoms or psychosocial risk factors.

## Introduction

**Figure 1.**
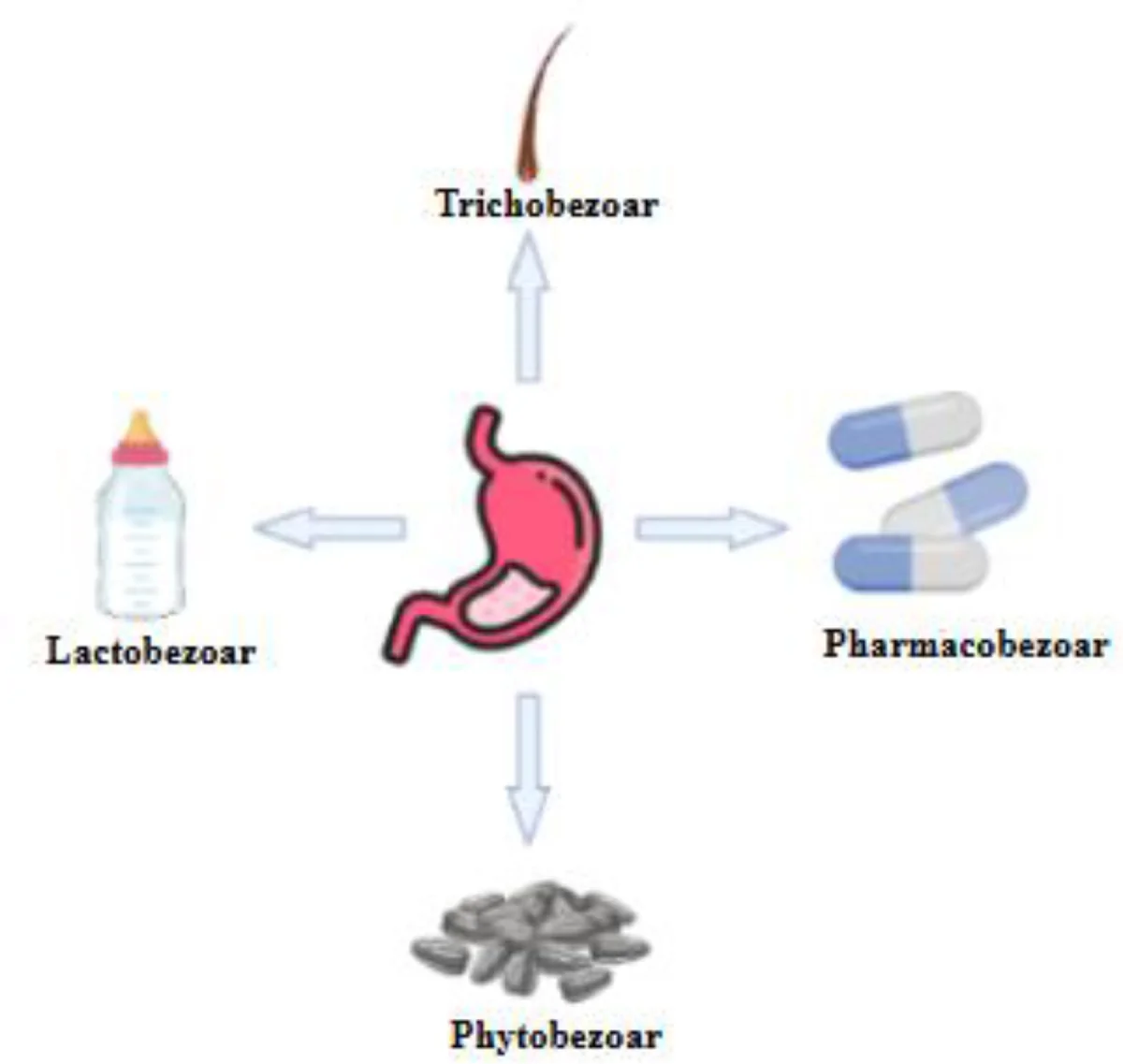
Types of bezoars. Source: authors. Created with Biorender^[^2–6,8^]^. Phytobezoar: composed of vegetable matter, it is the most common form. Trichobezoar: composed of hair. Pharmacobezoar: composed of ingested medications, usually extended-release capsules. Lactobezoar: composed of milk formulas.

Bezoars are conglomerates of indigestible materials within the gastrointestinal tract, primarily affecting the stomach but potentially causing obstruction at any digestive level^[^1,2^]^. The clinical presentation varies with the location and degree of obstruction, which may be partial or complete. Bezoars are classified according to the origin of the material; phytobezoars are the most frequent subtype (Fig. 1)^[^3–7^]^. This report presents a rare case of phytobezoar-induced ileal perforation in an adolescent, illustrating the diagnostic difficulties and management challenges associated with this entity. This case report has been reported in line with the SCARE checklist^[^7^]^.

HIGHLIGHTS

- Phytobezoar should be considered as a cause of small bowel obstruction in a patient with atypical abdominal pain and oral intolerance.
- The patient’s erratic clinical course, underscores the importance of a high index of suspicion for bezoars in cases of unexplained small bowel obstruction, when initial imaging is inconclusive.
- A multidisciplinary approach that addresses psychosocial factors is essential for accurate diagnosis and successful clinical outcomes, particularly in complex or atypical cases.

## Case presentation

A 17-year-old female presented to the emergency department of our center with a 2-week history of upper abdominal pain and emesis. Her social history was significant for a dysfunctional home environment and a chronic eating disorder characterized by persistent loss of appetite. Initial physical examination and biochemistry were unremarkable, leading to discharge.

**Figure 2.**
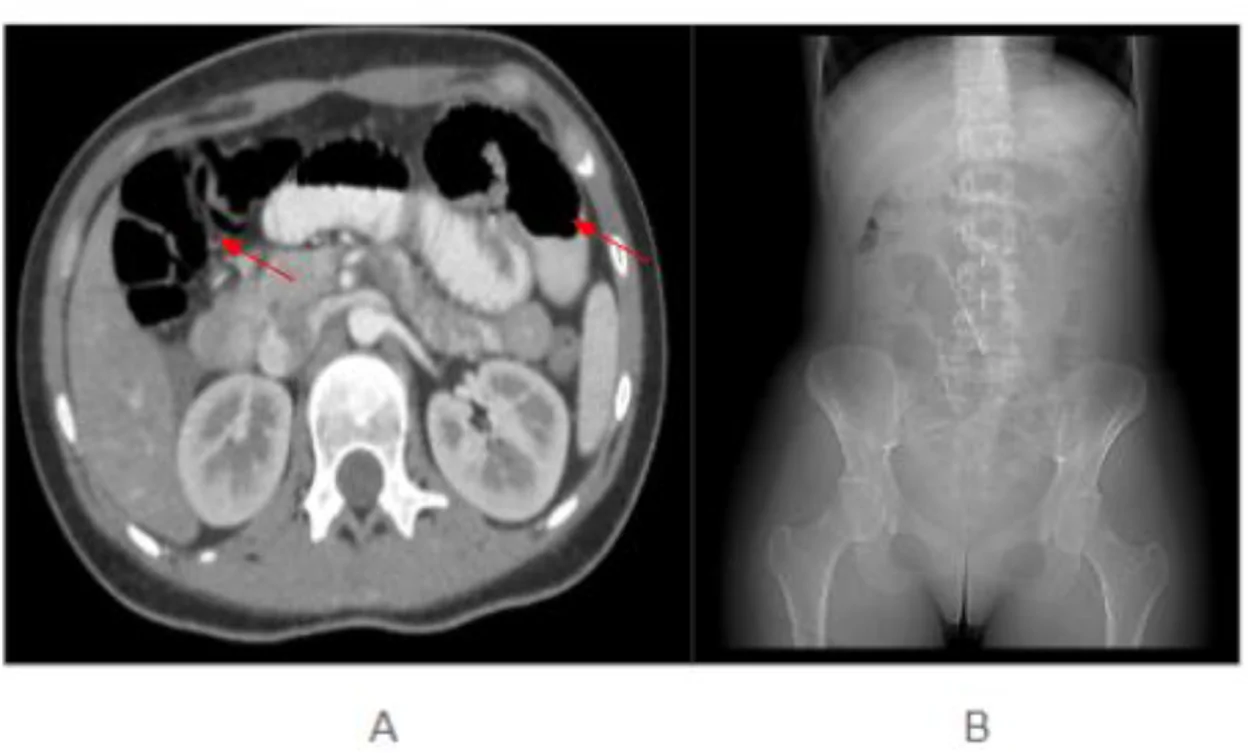
Computed tomography scan of the abdomen with intravenous contrast. (A) Transverse reconstruction. Arrows showing dilated intestinal loops. (B) Coronal reconstruction. Source: photographic images taken by the authors.

One week later, she was readmitted, presenting with vomiting and diffuse abdominal pain. Repeat blood tests and abdominal ultrasound were normal. Due to persistent symptoms, a contrast-enhanced abdominal CT scan was obtained, demonstrating distension of intestinal loops without transition zones or appendiceal inflammation (Fig. 2).

Despite persistent symptoms, there were no signs of a surgical abdomen. Management included nasogastric decompression and upper endoscopy, which revealed mild esophagitis and follicular gastritis, while colonoscopy was unremarkable. Porphyria was considered in the differential diagnosis; however, urinary porphobilinogen and 5-aminolevulinic acid levels were within normal limits.

Given the erratic clinical course, a diagnostic laparoscopy was performed but revealed no pathological findings. Suspecting inflammatory bowel disease, the gastroenterology team initiated mesalamine, hydrocortisone, and parenteral nutrition. Throughout her stay, the patient required continuous psychological support due to restricted affect and limited communication.

Conservative management was continued with close monitoring. Rheumatologic causes were also explored, but only complement consumption was found. A repeat CT scan showed intestinal distension but no mechanical obstruction. Despite a planned enteroscopy, the patient’s condition worsened, presenting generalized abdominal pain. Blood cultures grew methicillin-resistant *Staphylococcus aureus* (MRSA) and *Escherichia coli*, which led to initiation of broad-spectrum antibiotics. A bedside abdominal ultrasound revealed generalized peritonitis, prompting an emergency laparotomy.

**Figure 3.**
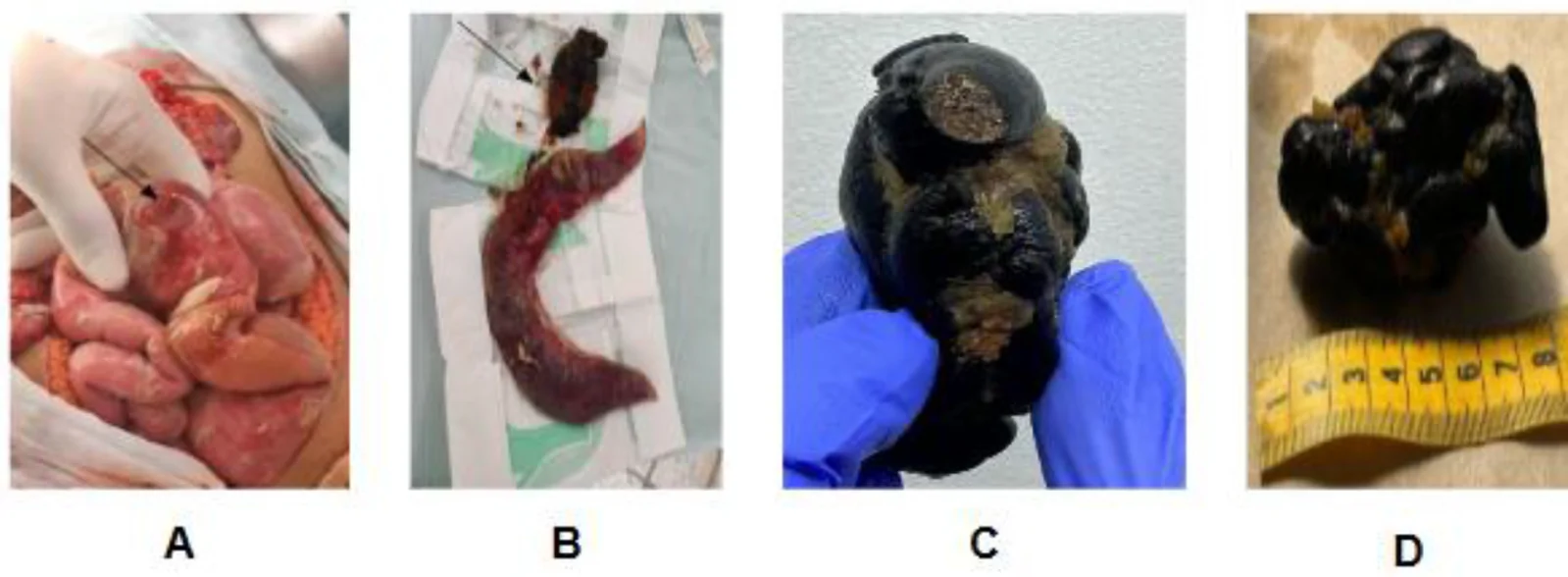
(A) Foreign body in the ileum. (B) Resection of 30 cm of the ileum. (C and D) Surgical specimen, 7 cm phytobezoar.

Intraoperatively, a perforation in the terminal ileum secondary to a foreign body was identified, associated with generalized peritonitis and abundant purulent fluid (Fig. 3). This required an extensive abdominal lavage, a 30 cm segmental ileal resection with primary anastomosis, prophylactic appendectomy, and management proceeded with an open abdomen technique.

Postoperatively, the patient gradually improved, allowing for vasopressor tapering, antibiotic de-escalation, and early mobilization. Following multidisciplinary rehabilitation and multimodal pain management, she was discharged with excellent clinical and psychosocial outcomes, transitioning to outpatient care.

## Discussion

The true incidence of bezoars remains unclear, although large retrospective series estimate that they account for 0.4-4% of mechanical intestinal obstructions ^[^9–11^]^. Phytobezoars, composed of indigestible vegetable matter, represent the most common subtype, accounting for approximately 40% of small bowel obstructions caused by bezoars^[^12^]^. In this case, the patient’s phytobezoar originated from *Inga edulis*, a fruit locally known in Colombia as “guama.”

**Figure 4.**
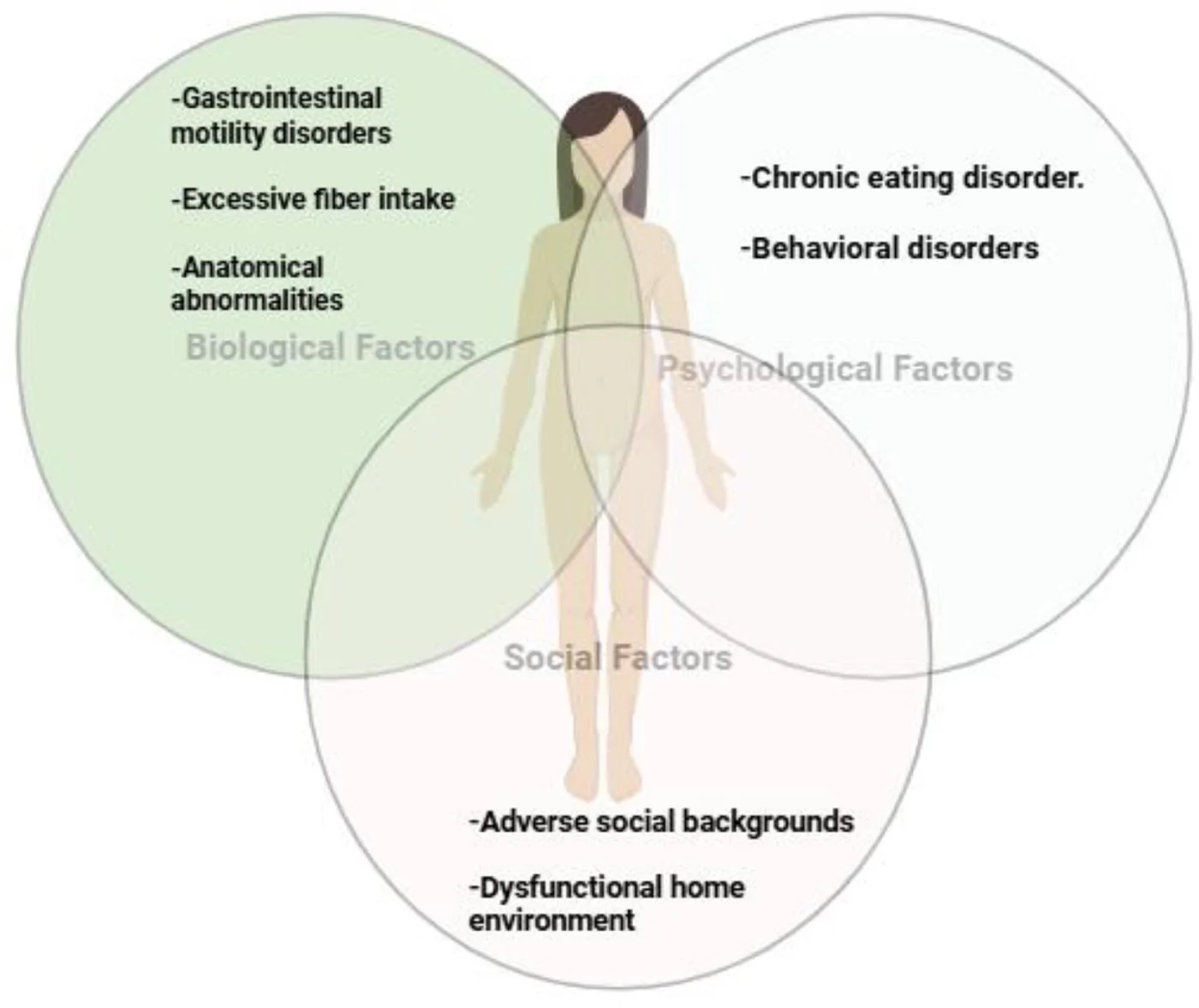
Integral diagnostic approach in phytobezoar. Created with Biorender ^[^11–15^]^.

This case is particularly noteworthy due to the adolescent patient’s atypical abdominal pain coupled with an adverse psychosocial history. Despite extensive diagnostic evaluations, including imaging and endoscopy, no definitive diagnosis was initially established. The unpredictable clinical course resulted in an urgent laparotomy, which ultimately identified the phytobezoar as the causative agent. The nonspecific and variable presentation of bezoars highlights the critical need for high clinical suspicion and an integral approach, especially in patients with predisposing clinical and psychosocial risk factors such as gastrointestinal motility disorders, psychiatric conditions, prior gastric surgery, excessive fiber intake, and adverse social backgrounds (Fig. 4)^[^12–15^]^.

Imaging studies are essential in the diagnostic approach, CT is considered the modality of choice due to its capacity to exclude other causes of intestinal obstruction. Radiological signs suggestive of phytobezoar include intestinal loop dilatation, bowel wall edema, and free intra-abdominal fluid ^[^14,1^]^. Some authors describe an ovoid intraluminal mass with a characteristic mottled gas pattern at the transition between dilated and collapsed loops as a pathognomonic sign, although this is not consistently observed. Owing to their radiolucent nature, phytobezoars may resemble fecal small bowel contents on CT, as demonstrated in this case, thereby complicating preoperative diagnosis ^[^14,15^]^.

Despite advanced imaging techniques, definitive diagnosis often relies on intraoperative findings. Awareness of this diagnostic challenge and consideration of clinical and psychosocial risk factors can improve early recognition.

## Conclusion

This case underscores the need to include bezoars in the differential diagnosis of patients with recurrent or persistent abdominal pain, particularly when the clinical course is inconsistent with the initial findings. Furthermore, underscores the importance of considering psychosocial factors and a multidisciplinary approach in patient care; these elements can significantly influence diagnostic accuracy and treatment outcomes.

## Research registration unique identifying number (UIN)

Name of the registry Comfamiliar Clinic Ethic Comitte 00136. The data that support the findings of this study are available from the corresponding authors on reasonable request
